# A novel magnetic compression technique for cystostomy in rabbits

**DOI:** 10.1038/s41598-022-16595-4

**Published:** 2022-07-16

**Authors:** Miaomiao Zhang, Jianqi Mao, Yixing Li, Shuqin Xu, Jingci Gai, Ting Lan, Yirui Wang, Yuxiang Ren, Aihua Shi, Yi Lyu, Xiaopeng Yan

**Affiliations:** 1 Department of Hepatobiliary Surgery, The First Affiliated Hospital of Xi’an Jiaotong University, 277 West Yanta Road, Xi’an, 710061 Shaanxi China; 2 National Local Joint Engineering Research Center for Precision Surgery & Regenerative Medicine, The First Affiliated Hospital of Xi’an Jiaotong University, 76 West Yanta Road, Xi’an, 710061 Shaanxi China; 3 Zonglian College, Xi’an Jiaotong University, Xi’an, Shaanxi China; 4 Department of Thoracic Surgery, The First Affiliated Hospital of Xi’an Jiaotong University, Xi’an, Shaanxi China; 5 Qide College, Xi’an Jiaotong University, Xi’an, Shaanxi China

**Keywords:** Urology, Urethra, Fistula creation

## Abstract

Magnetic compression technique (MCT) is a popular new anastomosis method. In this paper, we aimed to explore the feasibility of use of MCT for performing cystotomy in rabbits. The parent magnets and daughter magnets for rabbit cystostomy were designed and manufactured according to the anatomical characteristics of rabbit lower urinary tract. Twelve female New Zealand rabbits were used as animal models. After anesthesia, a daughter magnet was inserted into the bladder through the urethra, and the parent magnet was placed on the body surface projection of the bladder over the abdominal wall. The two magnets automatically attract each other. Postoperatively, the state of magnets was monitored daily, and the time when the magnets fell off was recorded. Cystostomy with MCT was successfully performed in all twelve rabbits. The mean operation time was 4.46 ± 0.75 min. The magnets fell off from the abdominal wall after a mean duration of 10.08 ± 1.62 days, resulting in the formation of bladder fistula. Macroscopic and microscopic examination showed that the fistula was well formed and unobstructed. The junction between bladder and abdominal wall was tight and smooth. We provide preliminary experimental evidence of the safety and feasibility of cystostomy based on MCT.

## Introduction

Cystostomy is a surgical operation for creation of a fistula to the bladder above the pubis, so that the urine is drained outside the body through the fistula. This procedure is most commonly performed to ameliorate the adverse effects of chronic urinary tract obstruction on the upper urinary tract or to ensure urinary tract healing after lower urinary tract surgery. Cystostomy is a routine operation in urological practice. Suprapubic cystostomy for percutaneous urinary drainage is a popular and effective treatment for patients with lower urinary tract symptoms due to bladder outlet obstruction or neurogenic bladder^1–3^. Percutaneous suprapubic cystostomy is now commonplace, and a variety of instruments have been developed to assist the surgeon^4^. Compared with open suprapubic cystostomy, percutaneous suprapubic cystostomy is a simple operation that entails lesser trauma and can be performed either at the bedside, in the clinic, or in the operating room^5^. As the bladder is an interperitoneal viscera, the anterior wall of the bladder is covered with membrane in some patients even when the bladder is full. Therefore, suprapubic cystostomy entails a risk of damage to the peritoneum and abdominal contents (bowel, vagina, uterus) in addition to other complications such as hematuria and wound infection^6^.

Magnetic compression technique (MCT) was first applied to perform microvascular anastomosis by Obora et al. in 1978^7^. Currently, it is a popular new anastomosis method. The MCT exploits the attraction between magnets to pull together an area of ischemic necrosis and its surrounding tissue to promote healing^8^. MCT has been applied in many scenarios, especially in the digestive system, such as esophageal magnamosis^9,10^, gastroesophageal magnamosis^11^, gastrojejunal magnamosis^12^, enteric magnamosis^13,14^, and choledochojejunostomy magnamosis^15,16^. In addition, MCT is also used for vascular anastomosis^17–19^, gastrostomy^20^, colostomy^21^, tracheoesophageal fistula^22^, and treatment of rectovaginal fistula^23^. In this study, we explored the feasibility of use of MCT to perform cystostomy in rabbits.

## Materials and methods

### Ethical statement

The research protocol and all experimental procedures were strictly in accordance with the Guidelines for the Care and Use of Experimental Animals issued by the Xi’an Jiaotong University Medical Center. This experimental study was approved by the Experimental Ethics Committee of the Xi’an Jiaotong University (Permit number: 2021-1536). In the previous preliminary experiments, we found that the urethra of female rabbits is easier to insert magnets than male rabbits, so we chose female rabbits as the animal model in this study. The study design in the manuscript strictly follows the recommendations in the ARRIVE guidelines.

### Animals

Twelve 4–6 months old female New Zealand rabbits, weighing 2.0–3.0 kg, were obtained from the Experimental Animal Center, College of Medicine, Xi’an Jiaotong University (Xi’an, China). The animal protocol was designed to minimize pain or discomfort to the animals. The animals were acclimatized to laboratory conditions (23 °C, 12 h/12 h light/dark, 50% humidity, ad libitum access to food and water) for one week prior to commencing the experiments. This was an exploratory study; therefore, all animals were included in the experimental investigations, and there was no control group. Intramuscular injection of pethidine hydrochloride (1 mg/kg) was administered every 12 h for analgesia for three days after surgery. Antibiotics are not used in experimental rabbits after surgery. At the end of the study, all animals were euthanized by a barbiturate overdose (intravenous injection, 60 mg/kg pentobarbital sodium) for tissue collection.

### Magnets

**Figure 1:**
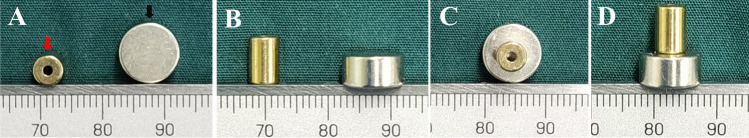
Photographs of the daughter magnet and the parent magnet. (**A**) Bottom view of daughter magnet (red arrow) and parent magnet (black arrow). (**B**) Side view of the magnets. (**C**) Bottom view of the two magnets attracted together. (**D**) Side view of the two magnets attracted together.

According to the anatomical characteristics of rabbit lower urinary tract, the parent and daughter magnets for cystostomy were designed and manufactured. We dissected female experimental rabbits and found that their urethra can be expanded to a maximum of about 6–7 mm, so the diameter of the daughter magnet is designed to be 5 mm. The daughter magnet was a cylinder (diameter: 5 mm; height: 7 mm) with a 1-mm hole in the center, and magnetic field intensity of 3680 GS. The parent magnet was a cylinder (diameter: 10 mm; length: 5 mm) with magnetic field intensity of 4260 GS (Fig. 1). The weight of parent magnet and daughter magnet was 2.470 g and 0.975 g, respectively. The parent and daughter magnets were made of neodymium-iron-boron (N45).

### Surgical procedure

**Figure 2:**
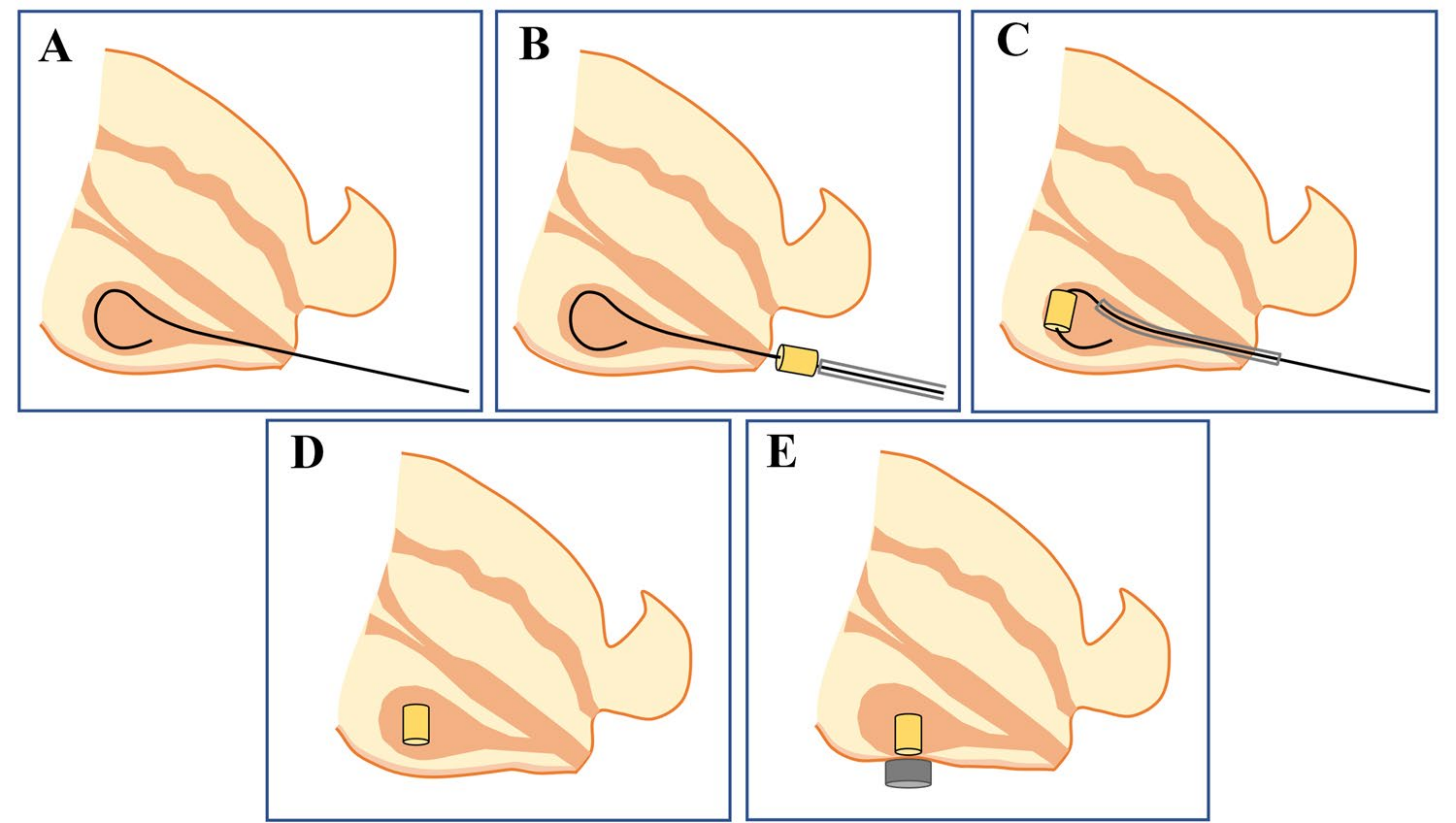
Schematic illustration of the surgical procedure. (**A**) The guide wire was inserted through the urethra into the bladder. (**B**) The daughter magnet and scalp needle tube were inserted through the end of the guide wire. (**C**) The daughter magnet was pushed along the guide wire into the bladder by pushing the scalp needle tube. (**D**) The guide wire and the scalp needle tube were removed. (**E**) After placing the parent magnet on the abdominal wall, the two magnets attract each other.

Before the surgery, rabbits were reared in single cage for 1 week and provided ad libitum access to standard rabbit chow and water. The rabbits were anesthetized by intravenous injection of 3% pentobarbital sodium (1 mL/kg) via ear vein after weighing. When the loss of paw retraction reflex was confirmed, the animal was placed in the supine position on the operating table and the limbs were immobilized. The lower abdomen and perineum were shaved. The interventional guide wire (0.035 in; TERUMO, Japan) was inserted through the urethra into the bladder and confirmed by X-ray (Fig. 2A). The end of the guide wire was inserted into the central hole of the daughter magnet and the scalp needle tube (Fig. 2B). By pushing the scalp needle tube, the daughter magnet was pushed along the guide wire into the bladder (Fig. 2C). Subsequently, the guide wire was removed and the daughter magnet was left in the bladder (Fig. 2D). The parent magnet was placed over the abdominal wall above the pubis so that the daughter magnet in the bladder was attracted to it (Fig. 2E). The daughter magnet was placed in the bladder according to the requirements of aseptic operation. The attachment of the magnets was monitored under X-ray, and the position of the magnets was confirmed by cystography.

### Calculation of operating time

The duration of operation was calculated as time elapsed from the beginning of insertion of the guide wire to the time when the parent and the daughter magnets were attracted together. The operation time of each rabbit was recorded.

### Postoperative care

After surgery, rabbits were housed individually in cages. The state of magnets was monitored daily, and the time of magnets falling off was recorded.

### Gross observation

After the magnets fell, the rabbits were euthanized with an overdose of anesthetic (intravenous injection, 60 mg/kg pentobarbital sodium). Urethra, bladder, and part of the abdominal wall around the site of cystostomy were taken as gross specimens to examine the fistula.

### Histological analysis

The cystostomy specimen was soaked overnight in 10% formalin. After fixation, the specimen was paraffin-embedded, and 4-mm thick sections were prepared from the anastomotic site. The sections were stained with hematoxylin and eosin and Masson trichromatic staining, and examined under a bright field microscope.

### Statistical analysis

SPSS statistical 18.0 software was used for data analysis. The quantitative data were described using mean ± standard deviation.

## Results

**Figure 3:**
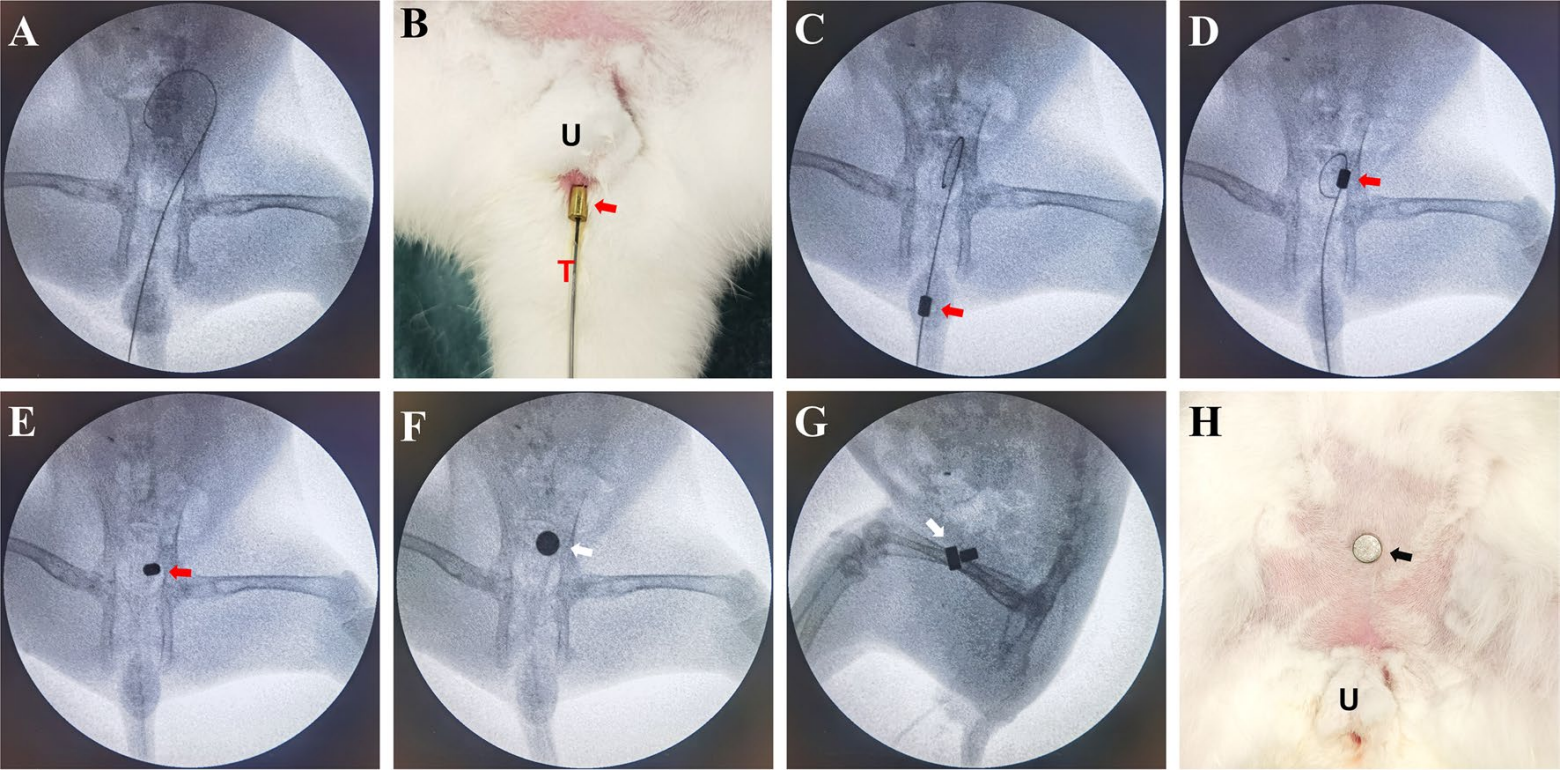
Representative intraoperative images. (**A**) X-ray image obtained after the guide wire had entered the bladder. (**B**) Daughter magnet (red arrow) is being inserted through the urethra (U) with the help of a scalp needle tube (T). (**C**–**E**) The daughter magnet is inserted into the bladder along the guide wire. (**F**) Anteroposterior radiograph showing the two magnets attracted together (white arrow). (**G**) Lateral radiograph showing the two magnets attracted together. (**H**) Photograph showing the parent magnet (black arrow) on the abdominal wall.

**Figure 4:**
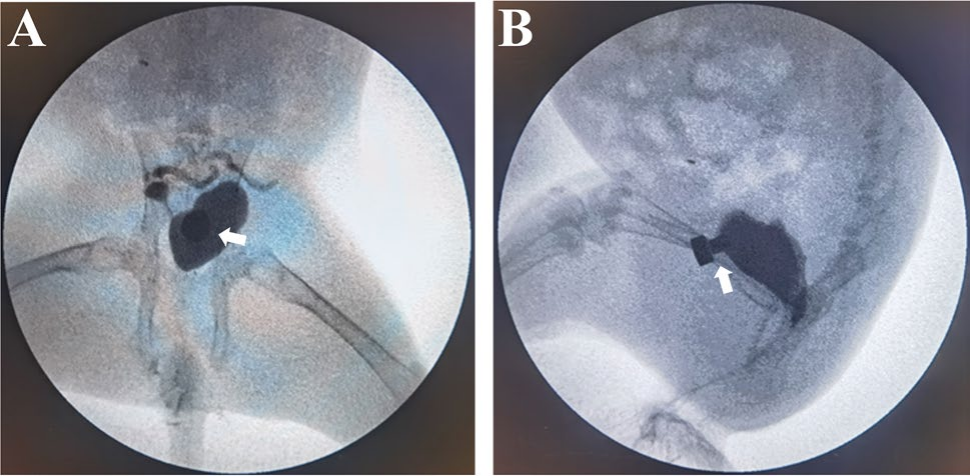
Confirmation of the position of the two magnets by cystography. (**A**) Anteroposterior radiograph; (**B**) Lateral radiograph.

**Figure 5:**
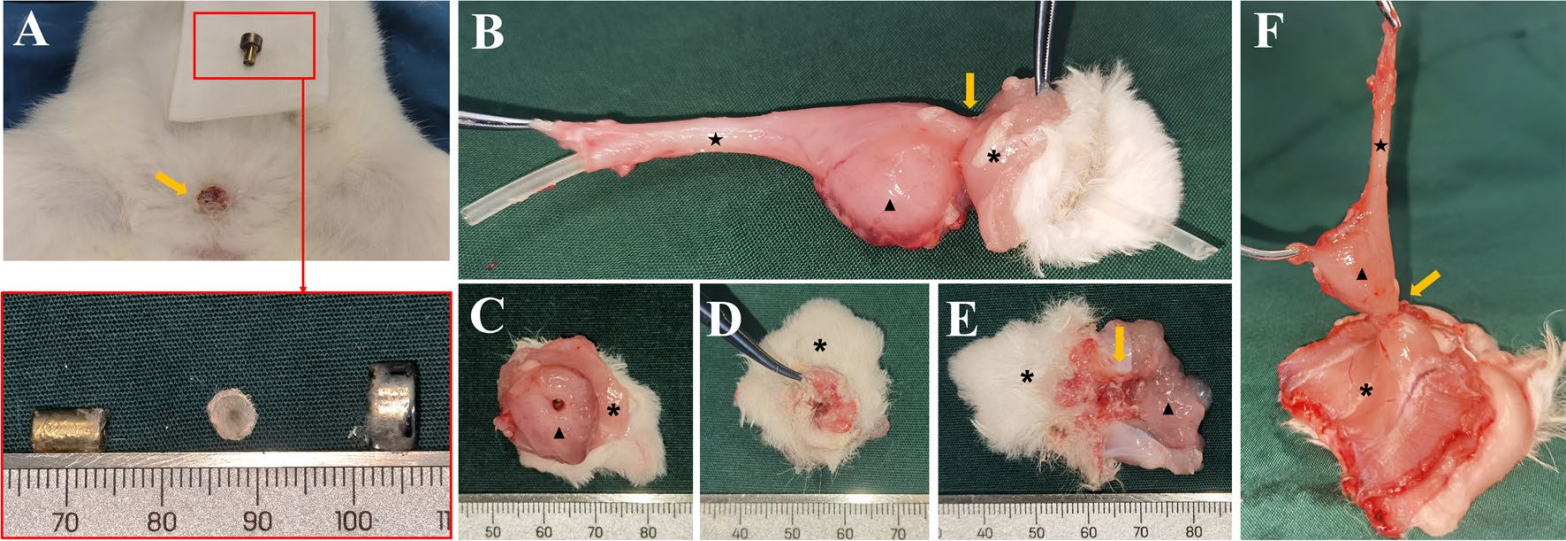
Gross specimens of bladder fistula. (**A**) Photograph showing the bladder fistula on the abdominal wall and the fallen-off magnets [the lower panel shows the daughter magnet (left), necrotic tissue (middle), and parent magnet (right)]. (**B**) The fistula specimen is unobstructed enough to allow the passage of the tube through it. (**C**) Fistula on the side of the bladder. (**D**) Fistula on the side of abdominal wall. (**E**) Longitudinal section through the fistula. (**F**) The bladder fistula is closely adhered to the abdominal wall, but there are no adhesions elsewhere (“★” is the urethra; “▲” is the bladder; “*” is the abdominal wall; the orange arrow indicates the fistula).

**Figure 6:**
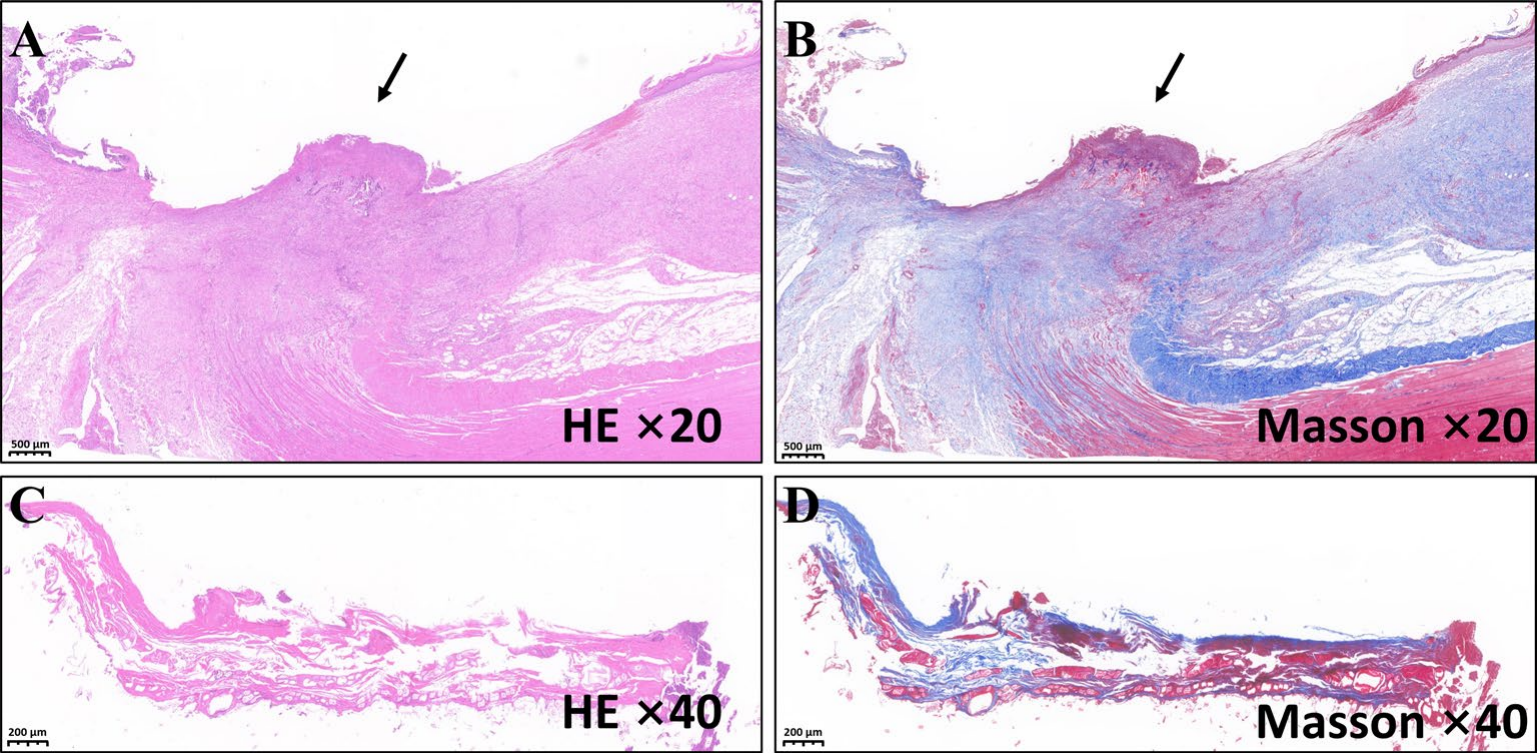
Histological specimens. (**A**,**B**) Anastomosis of bladder and abdominal wall. (**C**,**D**) Necrotic tissue between the parent and daughter magnet.

The daughter magnets were successfully inserted in the bladder in all animals, and the parent and daughter magnets were successfully attracted to each other. No gross hematuria was observed postoperatively. X-ray monitoring showed that the parent magnet and daughter magnet were attracted together (Fig. 3), and cystography showed that the magnets were in correct position (Fig. 4). The average operation time was 4.46 ± 0.75 min. All 12 rabbits survived with no postoperative complications such as bowel injury, hematuria, or wound infection. After a mean time of 10.08 ± 1.62 days after surgery, the magnets were detached from the abdominal wall, and the bladder fistula was formed. Necrotic bladder and abdominal wall tissues were seen between the detached daughter magnet and parent magnet (Fig. 5A). Macroscopic observation showed that the fistula was unobstructed (Fig. 5B–E), and the abdominal wall at the fistula was closely adhered to the bladder (Fig. 5F). There was no tissue adhesion in the pelvic cavity. Histological examination revealed a continuous and smooth mucosal surface at the junction of the abdominal wall with the bladder (Fig. 6).

## Discussion

In this experiment, cystostomy was successfully performed in 12 rabbits using MCT. The daughter magnet was successfully inserted into the bladder through the urethra. Intraoperative X-ray monitoring was used to confirm the position of the daughter magnets. The ostomy was successfully formed 8–13 days after the magnets were detached from the abdominal wall. There was close adhesion between the bladder and the abdominal wall at the site of fistula, but no adhesions were found in other parts of the pelvic cavity. The study provided preliminary evidence of the feasibility of performing cystostomy using MCT.

Magnetic compression anastomosis is a different mode of anastomosis compared to suture anastomosis and staple anastomosis. Magnetic compression anastomosis is a typical representative of "non-penetrating" anastomosis^24^. It has the characteristics of simple operation, reliable anastomosis, and wide application scenarios. Magnetic compression anastomosis is referred to as the third type of anastomosis after suture anastomosis and staple anastomosis. Magnetic compression anastomosis offers a unique advantage over other anastomosis methods in terms of its minimally invasive nature and suitability for anastomosis of complex structures; therefore, it is also called “smart anastomosis”. Theoretically, MCT can be used for the anastomotic reconstruction of all luminal organs of the body, but the contemporary research on magnetic compression anastomosis has largely focused on reconstruction of the digestive tract and vascular anastomosis^7,15^.

MCT can also be used for performing cystostomy. Ibrahim Uygun performed an experimental study using cylindrical magnets and spherical magnets to establish rat cystostomy animal models. Their results demonstrated the simplicity and safety of the operation^25^. However, it takes a long time to establish the cystostomy, which is related to the design of the magnet structure. When the spherical magnet and the cylindrical magnet are attracted, the contact surface is point-to-flat. This can lead to poor stability between the magnets and the tissue between the two magnets receives uneven compression. Therefore, we designed the cylindrical structure of the bladder fistula magnet. This design offers the following advantages: (1) There is a 1-mm diameter hole in the center of the daughter magnet, which allows the guide wire to pass through it, facilitating the insertion of the daughter magnet in the bladder. (2) The parent magnet is also designed with a cylindrical structure. The magnets attract each other to form a flat-to-flat force, which increases the stability between the parent magnet and the daughter magnet. (3) The bottom surface of the parent magnet outside the abdominal wall is larger than the bottom surface of the daughter magnet inside the bladder. This facilitates falling-off the parent and daughter magnets from the abdominal wall, and avoids the possibility of their falling into the bladder after the formation of the fistula. (4) After the cylindrical magnet is axially saturated and magnetized, a larger magnetic flux intensity can be obtained at both ends.

In this study, experimental rabbits were used as an animal model to verify the feasibility of MCT for cystostomy. However, due to the small number of experimental animals and the short observation time after the establishment of the fistula, further studies are required to assess the long-term outcomes of this technique. Experimental rabbits are quite different from the humans, so the next step is to use large animal models (dogs or pigs) to further optimize the operation path and observe the formation of the cystostomy. In this experimental design, cystostomy can only be established after the falling-off of the parent and daughter magnets. This is not suitable for clinical settings wherein patients typically require immediate establishment of an ostomy channel. For patients, magnet designed with a central hole can be used. When the parent and daughter magnets attract each other, the central hole of the magnet can be used to establish a channel immediately.

## Conclusions

Magnetic compression cystostomy is a potentially useful clinical application of MCT that represents an innovative alternative for conventional urological techniques. Magnetic compression cystostomy is a simple, minimally-invasive, and safe operation and has great potential for clinical application.
